# Assessment of knowledge, attitudes, pharmacotherapy counseling, and patient education about Down syndrome among community pharmacists: a cross-sectional study

**DOI:** 10.3389/fpubh.2026.1879369

**Published:** 2026-06-29

**Authors:** Anmar Al-Taie, Zekiye Yılmaz

**Affiliations:** 1 Clinical Pharmacy Department, Faculty of Pharmacy, Istinye University, Istanbul, Türkiye; 2 Department of Clinical Pharmacy, Faculty of Pharmacy, Acibadem Mehmet Ali Aydinlar University, Istanbul, Türkiye

**Keywords:** attitudes, community pharmacists, Down syndrome, knowledge, pharmacotherapy

## Abstract

**Background:**

Down syndrome (DS) is one of the most common causes of intellectual disability and is associated with multiple clinical challenges. The role of pharmacists in the care of individuals with DS is essential and cannot be overlooked, given the specific clinical characteristics that may affect the pharmacotherapy of this condition.

**Objective:**

This study aimed to assess community pharmacists’ knowledge and attitudes toward individuals with DS, as well as their provision of patient education and counseling in Türkiye.

**Methods:**

A descriptive cross-sectional study was conducted using an online questionnaire among community pharmacists in Türkiye.

**Results:**

A total of 497 community pharmacists participated in the study, of whom 77.7% had no prior DS training. Approximately 45.5% of participants demonstrated awareness of medications used in the pharmacotherapy of DS, and 39.6% were aware of potential adverse drug effects associated with medications used in individuals with DS. The mean scores were 5.4 ± 1.4 for general knowledge of DS, 5.7 ± 2.3 for DS treatment knowledge, and 5.1 ± 1.2 for attitudes toward DS. A statistically significant association was found between the knowledge of DS clinical characteristics (KDSC) and female gender (*p* = 0.01), higher educational level (*p* = 0.009), and prior DS training (*p* = 0.002).

**Conclusion:**

Turkish community pharmacists demonstrated a good level of knowledge and positive attitudes toward DS. However, gaps were identified in pharmacotherapy knowledge, as well as in patient education and counseling related to this condition.

## Introduction

Down syndrome (DS) is a genetic chromosomal disorder and the most common non-hereditary cause of intellectual disability. It is caused by trisomy of *Homo sapiens* chromosome 21 (HSA21) and affects approximately 1–2.2 per 1,000 live births worldwide (1, 2). The number of births with DS is approximately 1 in 530, based on data analysis by the Turkish Statistical Institute (3). DS is associated with numerous health problems and can affect several body systems, including the neurological, cardiovascular, and musculoskeletal systems. Congenital heart defects, cerebral hypoplasia, reduced neuronal density, muscle hypotonia, and intellectual disability are common among individuals with DS. In addition, several health conditions, such as hypothyroidism, autoimmune diseases, obstructive sleep apnea, epilepsy, hearing and vision impairments, hematological disorders, recurrent infections, anxiety disorders, and early-onset Alzheimer’s disease, occur more frequently in individuals with DS (4).

Social integration is an essential component of the wellbeing of individuals with disabilities. However, societal attitudes toward people with disabilities—which range from acceptance to stigmatization—are influenced by several factors, including cultural values, social norms, and public perceptions of their condition (5). Misconceptions about their personalities, behaviors, and developmental potential may contribute to negative attitudes and subsequent feelings of discomfort, pity, or rejection toward them (6). Inclusion has been shown to result in enhanced language and communication skills, improved social competence, and a higher quality of life for individuals with DS (7).

Previous studies have investigated public attitudes toward and knowledge of individuals with DS in a variety of settings (8–10). A majority of these studies were conducted among the general public and community members (6, 7, 9) or specific groups, such as parents and caregivers (8, 11, 12), educators, and teachers (13–16). However, to the best of our knowledge, few studies have evaluated the potential contributions and the practical role of community pharmacists in the care of individuals with DS in Türkiye.

Community pharmacists are among the few healthcare professionals who are readily accessible due to their convenient location and extended working hours. Consequently, they can play an active role in supporting the healthcare needs of individuals with DS, as they are well-positioned to assist patients, parents, and caregivers in various ways. These include providing information and educational support on the general characteristics, symptoms, and complications of DS, as well as other health conditions. Community pharmacists can also offer counseling on medication-related issues, including rational drug use, drug interactions, and adverse drug events, in an effective and meaningful manner (17–19). In Türkiye, community pharmacists are among the primary healthcare providers delivering pharmaceutical care services. Their responsibilities include prescription review, disease-related counseling, and educational support for medication-related problems, in addition to dispensing medications (20, 21). Therefore, this study aimed to assess community pharmacists’ knowledge and attitudes toward individuals with DS, including their knowledge of the condition and its pharmacotherapy, as well as their provision of patient education and counseling services in Türkiye.

## Methods

### Study setting

A cross-sectional descriptive study was conducted among community pharmacists between October and December 2024. Ethical approval for the study was obtained from the Healthcare Institutions, Medical Research Ethics Committee of Istinye University, Istanbul, Türkiye (Approval No. 23-293/2024/3).

### Study participants

This study included community pharmacists from across Türkiye. The study invitation and survey link were distributed to members of the Pharmacy Chambers through direct messages and e-mail. Participants were recruited using an exponential non-discriminative snowball sampling technique, with an anticipated response rate of approximately 50%. Assuming a 95% confidence level and a 5% margin of error, the minimum required sample size was calculated to be 345 participants. To compensate for potential non-response and missing data, the target sample size was increased by 15%, resulting in a required sample of 398 participants. Ultimately, 497 community pharmacists enrolled and completed all questionnaire items.

### Questionnaire setting

The study was conducted using a self-developed online questionnaire based on a comprehensive and in-depth review of the literature and designed in accordance with the objectives of this study (6–15, 22–24). The questionnaire underwent forward and backward bilingual translation from English into Turkish. In addition, the questionnaire items were evaluated for clarity, relevance, wording, and formatting. To further enhance the questionnaire’s linguistic accuracy and content validity, two academic experts reviewed the items and provided comments regarding face and content validity. The questionnaire was subsequently revised based on their recommendations. Following this process, a pilot study was conducted among 15 community pharmacists who were not included in the final study sample. Feedback obtained during the pilot phase was used to improve the clarity and comprehensibility of the questionnaire items.

Google Forms was used as the web-based platform for survey distribution. The questionnaire link was distributed via direct messaging and e-mail, accompanied by detailed information about the study. To increase the response rate, participants were encouraged to share the survey link with other community pharmacists. Before participation, all respondents were provided with information regarding the study objectives and procedures and were required to provide informed consent electronically. Each participant completed an electronic consent form before accessing the questionnaire. Several verification measures were implemented to prevent duplicate responses and ensure that each participant completed the survey only once. Participants were assured of the anonymity and confidentiality of their responses and informed that participation was entirely voluntary. The questionnaire was designed to require approximately 15 min to complete.

### Study variables and outcomes

The questionnaire consisted of four sections. The first section collected participants’ demographic characteristics. The second section assessed knowledge regarding Down syndrome (KDS) and comprised 30 items distributed across four domains: general knowledge about DS (GKDS), knowledge of DS screening (KDSS), knowledge of DS clinical characteristics (KDSC), and knowledge of DS treatment (KDST). Cronbach’s alpha was used to assess internal consistency, which sufficiently demonstrated the scale’s reliability and validity (*α* = 0.783). The third section assessed attitudes toward individuals with DS (ADS) and consisted of six items. Cronbach’s alpha was used to assess internal consistency, which sufficiently demonstrated the scale’s reliability and validity (*α* = 0.701). Items in both the knowledge and attitude sections were evaluated using three response options: “Agree,” “Disagree,” and “Do not know.” Responses were scored according to their correctness, with correct answers assigned a score of 1 and incorrect or “Do not know” responses were assigned a score of 0. The total knowledge score was calculated by summing the scores of all knowledge items, with higher scores indicating good knowledge. Similarly, higher attitude scores reflected positive attitudes toward individuals with DS. Total knowledge scores were dichotomized into good and poor or insufficient knowledge using a cutoff of 15 out of 30; scores ≥15 were classified as good knowledge, whereas scores <15 were classified as poor or insufficient knowledge. Similarly, total attitude scores were dichotomized into positive and negative attitudes using the cutoff of 3/6; scores ≥3 were classified as positive knowledge, whereas scores <3 were classified as negative attitudes. The fourth section evaluated community pharmacists’ knowledge and practices related to DS treatment and patient counseling. This section consisted of eight items addressing various aspects of pharmaceutical care, including the provision of appropriate medication instructions and counseling for patients with DS and their family members or caregivers. Responses in this section were analyzed and reported based on the proportion of correct answers provided by participants.

### Statistical analysis

The Statistical Program for Social Science Research, edition 23.0, and Microsoft Office Excel 2013 were used for analyzing the data. Descriptive statistics, including frequencies, percentages, means, and standard deviations (SDs), were used to summarize the demographic characteristics of the participants. A one-way ANOVA was used to compare mean knowledge and attitude scores across demographic groups, including variables such as age, years of experience, and educational level. An independent samples t-test was used to compare two groups, and a one-way ANOVA (*F*-test) was used to compare three or more groups. Statistical significance was set at a *p*-value of <0.05, with a 95% confidence interval.

## Results

A total of 497 community pharmacists were included in the study, with a mean age of 32.0 ± 13.96 years. The majority of the participants were female (59.4%). Less than half of the respondents (42.6%) had fewer than 5 years of pharmacy practice experience. Regarding educational qualifications, 79.9% of the participants held a bachelor’s degree in pharmacy. A majority of community pharmacists (77.7%) reported having received no previous training on how to interact with or care for individuals with DS, as shown in Table 1.

**Table 1:** Demographic characteristics of the study participants.

Variables		*N* = 497
		*n* (%)
Sex	Female	295 (59.4)
	Male	202 (40.6)
Age (years)	20–29	228 (45.9)
	30–39	113 (22.7)
	40–49	60 (12.1)
	50–59	46 (9.3)
	≥60	50 (10)
Duration of pharmacy experience (years)	<5	212 (42.6)
	5–10	91 (18.3)
	11–20	68 (13.7)
	>20	126 (25.4)
Pharmacy educational degree	Bachelor’s degree	397 (79.9)
	Master’s degree	86 (17.3)
	Doctorate degree	14 (2.8)
Having training course(s) about DS	Yes	111 (22.3)
	No	386 (77.7)
Learning lines about DS	Healthcare workers	95 (19.1)
	Internet	80 (16.1)
	Literature	201 (40.4)
	Media	28 (5.6)
	Family/friends/relatives	93 (18.7)

Data presented as number (*n*) and percentage (%); DS, Down syndrome.

Table 2 presents the participants’ knowledge regarding DS. Overall, a good level of general knowledge about DS (GKDS) was observed among the participants. A majority of respondents correctly identified that “Down syndrome is a genetic disorder” (88.5%), that “parents have a chance of having a child with Down syndrome if one parent has Down syndrome or carries a genetic abnormality associated with the condition” (80.2%), and that “adults with Down syndrome can get married and have children” (61.6%). Regarding knowledge of DS screening (KDSS), a high proportion of participants correctly recognized that DS can be detected during pregnancy (92.2%). In addition, 65.4 and 53.3% of participants correctly responded to the statements concerning the need for DS testing before marriage and the possibility of detecting DS through premarital blood testing, respectively. With respect to the KDSC, a majority of participants demonstrated good knowledge regarding the impact of DS on daily social functioning (78.2%) and the fact that individuals with DS may develop various health complications (74.1%). Regarding knowledge of DS treatment (KDST), a majority of participants correctly disagreed with the statements that DS is a curable disease (81.7%), that it can be treated by blood transfusion (68.6%), and that it can be treated with medications (74.5%). In addition, 44.9% correctly disagreed with the statement that DS requires a specific curative treatment.

**Table 2:** Knowledge of community pharmacists about Down syndrome.

Questions	Agree	Disagree	Do not know
	*n* (%)	*n* (%)	*n* (%)
Domain 1: General knowledge regarding Down syndrome (GKDS)			
Down syndrome is a genetic and inherited disease	440 (88.5)	26 (5.2)	31 (6.3)
Down syndrome is a condition that can be transmitted from one person to another, like infections	18 (3.6)	470 (94.6)	9 (1.8)
Incorrect nutrition or a poor diet is an important risk factor for Down syndrome	137 (27.6)	271 (54.5)	89 (17.9)
Marriage between close relatives can increase the risk of Down syndrome	443 (89.1)	19 (3.8)	35 (7.1)
A normal person can marry *an individual with Down syndrome*	262 (52.7)	91 (18.3)	144 (29)
The parents have a chance of having a child with Down syndrome if one parent is a Down syndrome carrier or has the Down syndrome gene	399 (80.2)	52 (10.5)	46 (9.3)
Adults with Down syndrome can get married and have babies	306 (61.6)	84 (16.9)	107 (21.5)
Pregnancy should be aborted if the fetus is diagnosed with Down syndrome	115 (23.1)	224 (45.1)	158 (31.8)
Domain 2: knowledge regarding Down syndrome screening (KDSS)			
Down syndrome can be detected by a blood test	311 (62.6)	82 (16.5)	104 (20.9)
Down syndrome can be detected by taking blood samples of both partners before marriage	260 (52.3)	113 (22.7)	124 (25)
Down syndrome test is necessary/compulsory before marriage	325 (65.4)	89 (17.9)	83 (16.7)
Down syndrome can be detected during pregnancy	458 (92.2)	3 (0.6)	36 (7.2)
Domain 3: knowledge regarding Down syndrome clinical characteristics (KDSC)			
Individuals with Down syndrome are as healthy as normal individuals	301 (60.6)	109 (21.9)	87 (17.5)
Individuals who are Down Syndrome carriers have problems in their day-to-day social life	123 (24.7)	291 (58.6)	83 (16.7)
Individuals with Down syndrome have problems in their day-to-day social life	389 (78.2)	52 (10.5)	56 (11.3)
Individuals with Down syndrome may not have symptoms of the disease	131 (26.3)	227 (45.7)	139 (28)
Individuals with Down syndrome are always suffering from intellectual disability	149 (30)	238 (47.9)	110 (22.1)
Individuals who are Down Syndrome carriers have a higher risk of infection and/or illnesses	150 (30.1)	211 (42.5)	136 (27.4)
Individuals with Down syndrome have a higher risk of infection and/or illnesses	316 (30.6)	70 (14.1)	111 (22.3)
Individuals with Down syndrome are more likely to develop symptoms or complications related to the blood, hearing and vision, and heart	368 (74.1)	34 (6.8)	95 (19.1)
Domain 4: knowledge regarding Down syndrome treatment (KDST)			
Individuals who are Down Syndrome carriers can survive if Down Syndrome is not treated	419 (84.3)	20 (4)	58 (11.7)
Individuals with Down syndrome can survive if Down Syndrome is not treated	339 (68.2)	61 (12.3)	97 (19.5)
Down syndrome is a preventable disease	126 (25.3)	247 (49.7)	124 (25)
Individuals who are Down Syndrome carriers should avoid some specific foods	138 (27.8)	188 (37.8)	171 (34.4)
Individuals with Down syndrome should avoid some specific foods	261 (52.5)	92 (18.5)	144 (29)
Down syndrome is a completely curable or treatable disease	26 (5.2)	406 (81.7)	65 (13.1)
Down syndrome can be treated with a blood transfusion	26 (5.2)	341 (68.6)	130 (26.2)
Individuals who are Down Syndrome carriers need a specific treatment	135 (27.1)	223 (44.9)	139 (28)
Individuals with Down syndrome need a specific treatment	277 (55.7)	104 (21)	116 (23.3)
Down syndrome can be treated with medications	34 (6.8)	370 (74.5)	93 (18.7)

Data presented as number (*n*) and percentage (%).

Similarly, the majority of the participants demonstrated positive attitudes toward individuals with DS, as shown in Table 3. The majority of respondents agreed with the need for more specialized healthcare services for individuals with DS (93.4%) and supported increasing public awareness of DS through various media platforms, including electronic and print media (95.6%). The mean attitude score was 5.1 ± 1.2.

**Table 3:** Community pharmacists’ attitudes toward Down syndrome.

Questions	Agree	Disagree	Do not know
	*n* (%)	*n* (%)	*n* (%)
Is it possible that children with Down syndrome understand what we say when we talk to them?	433 (87.1)	12 (2.4)	52 (10.5)
Would you recommend for them special help/health services?	464 (93.4)	10 (2)	23 (4.6)
As an employer, would you consider employing an individual with Down syndrome	358 (72)	89 (18)	50 (10)
Would you like to play your role in improving the quality of life of individuals with Down syndrome?	416 (83.7)	9 (1.8)	72 (14.5)
Would you support individuals with Down syndrome in living a normal life?	445 (89.5)	11 (2.2)	41 (8.3)
The government should use electronic or print media to raise awareness of Down Syndrome	475 (95.6)	5 (1)	17 (3.4)

Data presented as number (*n*) and percentage (%).

Regarding community pharmacists’ knowledge of DS, the mean scores for the different knowledge domains were as follows: general knowledge of DS (5.4 ± 1.4), knowledge of DS screening (2.7 ± 1.1), knowledge of DS clinical characteristics (4.1 ± 1.4), and knowledge of DS treatment (5.7 ± 2.3). Community pharmacists also demonstrated positive attitudes toward individuals with DS, with a mean attitude score of 5.1 ± 1.2, as shown in Table 4.

**Table 4:** Rate of knowledge and attitudes of community pharmacists about Down syndrome.

Domains of the KCAHW questionnaire	Mean ± SD
Domain 1: General knowledge regarding Down syndrome	5.4 ± 1.4
Domain 2: Knowledge regarding Down syndrome screening	2.7 ± 1.1
Domain 3: Knowledge regarding Down syndrome clinical characteristics	4.1 ± 1.4
Domain 4: Knowledge regarding Down syndrome treatment	5.7 ± 2.3
*Community pharmacists’ attitudes toward Down syndrome*	5.1 ± 1.2

SD, standard deviation.

The mean and standard deviation values were calculated from the participants’ responses to each single-scale items.

The association between participants’ total questionnaire scores and demographic characteristics showed statistically significant differences in KDSC with female gender (*p* = 0.01), higher educational level (*p* = 0.009), and prior DS training (*p* = 0.002). Significant differences in knowledge of DS treatment (KDST) were observed among participants aged ≥60 years (p = 0.002) and those with more than 20 years of pharmacy practice experience (*p* = 0.001). In addition, knowledge of DS screening (KDSS) was significantly associated with prior DS training (*p* = 0.01), as shown in Table 5. However, the association between participants’ total attitude scores and demographic characteristics showed a statistically significant difference only with female gender (*p* = 0.004), as shown in Table 6.

**Table 5:** Association between the KDS score and demographic characteristics.

Variables		GKDS	*p*-value	KDSS	*p*-value	KDSC	*p*-value	KDST	*p*-value
Gender	Male	5.5 ± 1.5	0.33	2.7 ± 1.2	0.81	3.9 ± 1.6	0.01	5.4 ± 2.4	0.24
	Female	5.3 ± 1.4		2.7 ± 1.1		4.2 ± 1.4		5.8 ± 2.1	
Age (years)	20–29	5.2 ± 1.5	0.05	2.6 ± 1.2	0.13	3.9 ± 1.6	0.36	5.3 ± 2.4	0.002
	30–39	5.6 ± 1.4		2.9 ± 1.0		4.2 ± 1.3		5.8 ± 1.96	
	40–49	5.5 ± 1.4		2.8 ± 1.1		4.2 ± 1.5		5.5 ± 2.1	
	50–59	5.4 ± 1.1		2.8 ± 1.0		4.1 ± 1.2		6.4 ± 2.0	
	≥ 60	5.7 ± 1.2		2.8 ± 1.0		4.4 ± 1.4		6.5 ± 2.4	
Duration of pharmacy experience (years)	< 5	5.2 ± 1.5	0.10	2.6 ± 1.2	0.07	3.9 ± 1.6	0.21	5.3 ± 2.4	0.001
	5–10	5.4 ± 1.4		2.7 ± 1.0		4.1 ± 1.3		5.7 ± 1.9	
	11–20	5.6 ± 1.4		2.8 ± 1.1		4.1 ± 1.4		5.8 ± 1.0	
	20	5.5 ± 1.2		2.9 ± 1.0		4.3 ± 1.3		6.2 ± 1.1	
Pharmacy educational degree	Bachelor’s degree	5.3 ± 1.4	0.23	2.7 ± 1.1	0.53	3.9 ± 1.4	0.009	5.6 ± 2.3	0.17
	Master’s degree	5.6 ± 1.3		2.8 ± 1.1		4.6 ± 1.4		6.1 ± 2.2	
	Doctorate degree	5.3 ± 1.5		2.7 ± 1.0		4.1 ± 1.6		5.5 ± 2.5	
Having training course (s) about Down syndrome	Yes	5.5 ± 1.4	0.24	2.9 ± 1.0	0.01	4.5 ± 1.2	0.002	5.9 ± 2.4	0.10
	No	5.4 ± 1.4		2.6 ± 1.1		3.9 ± 1.5		5.6 ± 2.2	

Significant at *p* < 0.05.

**Table 6:** Association between community pharmacists’ attitudes toward Down syndrome score and demographic characteristics.

Variables		ADS	*p*-value
Gender	Male	4.9 ± 1.4	0.004
	Female		
		5.2 ± 1.0	
Age (years)	20–29	5.1 ± 1.3	0.93
	30–39	5.0 ± 0.9	
	40–49	5.0 ± 1.1	
	50–59	5.1 ± 1.0	
	≥60	5.1 ± 1.1	
Duration of pharmacy experience (years)	<5	5.1 ± 1.4	0.94
	5–10	5.0 ± 0.9	
	11–20	5.1 ± 1.0	
	20	5.1 ± 1.1	
Pharmacy educational degree	Bachelor’s degree	5.0 ± 1.2	0.15
	Master’s degree	5.8 ± 0.9	
	Doctorate degree	5.5 ± 0.5	
Having training course (s) about Down syndrome	Yes	5.2 ± 0.9	0.27
	No	5.0 ± 1.2	

Significant at *p* < 0.05.

**Figure 1:**
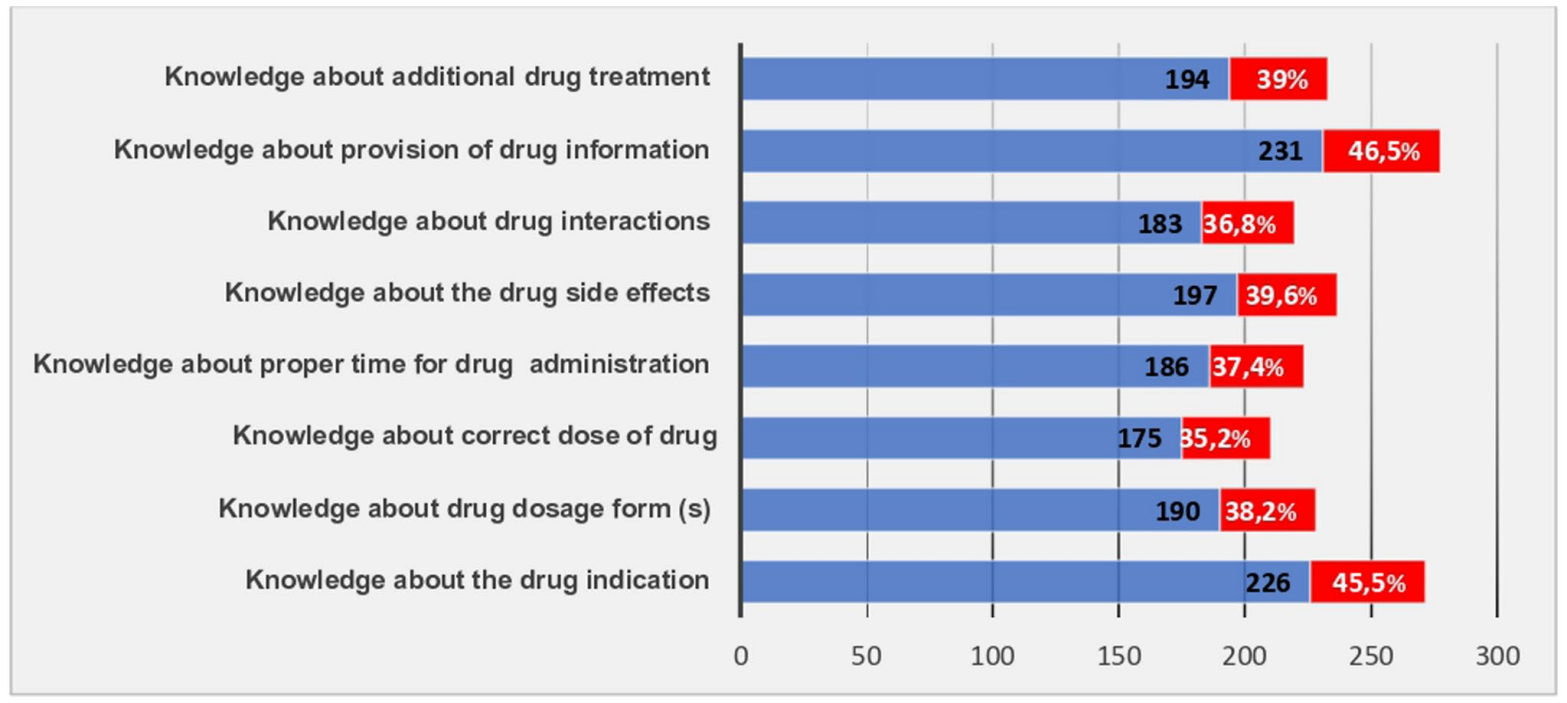
Knowledge of community pharmacists about pharmacotherapy of Down syndrome. Data presented as number (*n*) and percentage (%). The blue color represented the frequency (*n*), and the red color represented the percentage (%). The responses were reported based on the correct answering of these questions.

Community pharmacists reported relatively insufficient knowledge regarding the principles of DS pharmacotherapy in several areas, including drug indications (45.5%) and dosage forms used in DS management (38.2%). Similarly, limited knowledge was observed regarding drug side effects (39.6%), provision of appropriate medication information (46.5%), appropriate timing of drug administration (37.4%), and correct drug dosing (35.2%). Additional findings on pharmacists’ knowledge of DS pharmacotherapy are shown in Figure 1.

## Discussion

Down syndrome is one of the leading causes of intellectual disability and neurodevelopmental disorders, and the affected individuals experience a wide range of health challenges. The importance of community pharmacists having adequate knowledge of DS and its management is considerable, given their role in providing care and support for various health conditions, including this genetic disorder and its associated comorbidities. This study aimed to evaluate community pharmacists’ knowledge and attitudes toward individuals with DS in Türkiye. Previous studies in the literature have assessed public knowledge, awareness, and attitudes toward individuals with DS in a variety of populations, including the general public and community members (7–9), teachers and educators (13–16), parents and caregivers (8, 11), and schoolchildren (22, 23). However, few studies have examined the knowledge and role of community pharmacists in relation to individuals with DS. In the present study, the majority of community pharmacists demonstrated a good level of general knowledge regarding individuals with DS. This was reflected in participants’ responses to the GKDS domain and was supported by a relatively high mean GKDS score. These findings are consistent with a previous study by Rabbani et al. (24), which reported positive knowledge regarding DS among medical and health sciences students.

A crucial component of prenatal care is serum screening for DS. Healthcare professionals should ensure that women recommended for DS screening are informed about the condition, the implications of screening tests, and what it means to have a child with DS. In general, awareness of the condition being screened for influences participation in DS screening tests (25, 26). Regarding knowledge of DS screening, the majority of participants in our study reported a good level of knowledge about the importance of detecting DS during pregnancy, which was reflected in a relatively high mean KDSS score. The findings of our study are consistent with those of Rabbani et al. (24), who reported that a majority of the healthcare participants recognized prenatal screening as essential for detecting DS. However, the findings of the current study contrast with those reported by Chilaka et al. (26), who assessed women’s knowledge of DS during antenatal screening. The study identified a lack of knowledge and awareness of DS among women, contradicting the assumption that parents are adequately informed by the time they consult healthcare providers. Similarly, a cross-sectional study conducted in Jeddah, Saudi Arabia, to assess knowledge and attitudes toward DS in the general population, reported a low knowledge of DS screening, with 19% of participants believing that DS can be diagnosed based on ultrasound and blood tests (27). In many countries, antenatal DS screening is currently a standard procedure. Both the benefits and risks of screening should be clearly communicated to prospective parents. Previous studies have shown that many women decline DS screening during pregnancy and that a substantial proportion underestimate the associated risks (28, 29). In Türkiye, a national prenatal screening program for DS is in place to detect congenital anomalies before birth (30). In the present study, community pharmacists demonstrated a good level of knowledge regarding the clinical features of DS, including its impact on daily social functioning and the increased susceptibility of individuals with DS to various complications. These positive findings were reflected in a relatively high mean KDSC score. Studies in the literature are also consistent with our findings regarding knowledge of the clinical manifestations of DS (8, 24).

Overall, the majority of community pharmacists in our study demonstrated positive attitudes toward individuals with DS, which was reflected in a relatively high mean attitude score. These findings may be attributed to the possibility that respondents received education on genetic disorders during their undergraduate and/or postgraduate training. In this regard, education is expected to enhance healthcare providers’ knowledge and awareness of genetic conditions, such as DS, thereby improving early detection and intervention. In comparison with previous studies assessing healthcare professionals’ attitudes toward individuals with DS, Uysal et al. (31), who conducted a study among nursing students, reported moderate attitudes toward individuals with disabilities. Additionally, Hall and Hollin (32) examined the effect of an educational session on medical students’ attitudes toward DS and found a significant improvement following the intervention. Similarly, Rabbani et al. (24), who conducted a study among medical and health sciences students, reported positive attitudes toward individuals with DS. However, these findings contrast with those of Alhaddad et al. (27), who reported overall poor attitudes among participants (47%), and Ahmad and Saad (33), who found inadequate knowledge, awareness, and attitudes regarding individuals with DS among university students.

In the present study, statistically significant differences were observed in participants’ KDSC and attitude total scores with respect to female gender. KDST total scores were significantly associated with age ≥60 years, higher educational level was associated with KDSC, and KDST total scores were associated with more than 20 years of pharmacy practice experience. In addition, KDSS total scores were significantly associated with prior DS training. These findings are consistent with previous studies that have identified several factors influencing knowledge and perceptions of individuals with disabilities, including age, gender, and educational level (22, 23, 31–33). In our study, higher educational level was significantly associated with greater knowledge of DS, as individuals with higher education are generally expected to have greater knowledge and make stronger contributions to the early detection and management of genetic disorders (34). Similarly, age was positively associated with good knowledge of DS, which may be explained by the fact that community pharmacists become more aware of this genetic disorder with increasing age and years of professional experience. Nonetheless, inconsistent findings have been reported by Uysal et al. (31) and Ahmad et al. (33), suggesting that student attitudes toward individuals with DS and disabilities are negatively associated with age. Meanwhile, the female gender was significantly associated with a good knowledge of DS. This finding is consistent with a previous study showing that knowledge and attitudes toward individuals with DS are significantly influenced by female gender (24). Years of pharmacy practice experience were also significantly associated with the level of knowledge of DS; this may be explained by the fact that, as pharmacists gain experience, they become more exposed to and knowledgeable about a wider range of health conditions, including genetic disorders, such as DS.

Individuals with DS frequently present with multiple comorbidities that require complex pharmacotherapeutic management. Individuals with DS also have a high drug utilization rate and may respond differently to certain pharmacological therapies, thereby providing optimal care. Consequently, individuals with DS are often exposed to polypharmacy and significant healthcare expenses, which increase the risk of drug–drug interactions, adverse drug reactions, and medication-related problems (35, 36). Another important challenge relates to altered pharmacokinetics and pharmacodynamics among individuals with DS. Previous studies have suggested that genetic and physiological alterations associated with trisomy 21 may influence drug metabolism, drug disposition, and therapeutic response. Increased susceptibility to adverse drug reactions has been reported with several medications. Therefore, enhanced therapeutic monitoring and individualized dose adjustment may be necessary in this population (35).

Meanwhile, inadequate living arrangements and the intellectual disability linked to DS may be risk factors for poor medication adherence. Medication adherence also represents a significant challenge in patients with DS. Intellectual disability, communication difficulties, dependence on caregivers, and limited health literacy may affect the patient’s ability to understand and follow medication instructions correctly. In many cases, caregivers play a central role in medication administration and monitoring, emphasizing the need for clear counseling and continuous patient and caregiver education by healthcare professionals, particularly pharmacists (35–37). As life expectancy in individuals with DS continues to improve, healthcare providers increasingly encounter DS-related issues. This transition further complicates pharmacotherapy management and highlights the importance of multidisciplinary care, particularly involving pharmacists, who can contribute significantly through medication review, adherence support, identification of drug-related problems, and patient counseling. Therefore, monitoring for adverse effects, adherence, and treatment efficacy is crucial and would contribute to improving clinical care in this population (35, 38).

In the present study, community pharmacists reported insufficient knowledge regarding the pharmacotherapy of DS in several areas, including medication indications and dosage forms used in the management of DS. Similarly, limited knowledge was reported regarding drug side effects, provision of appropriate medication information, appropriate timing of drug administration, and correct drug dosing.

Previous studies have demonstrated that pharmacist involvement is an essential component of patient care, contributing to improved medication adherence and reduced drug-related problems, such as adverse drug events and drug interactions, thereby enhancing patients’ quality of life (17–19). Nonetheless, additional strategies should be implemented to improve community pharmacists’ knowledge of DS and its pharmacotherapy, including participation in conferences, training programs, and interprofessional education and collaborative practice activities.

### Strengths and limitations of the study

To our knowledge, this is the first cross-sectional study to evaluate community pharmacists’ knowledge and attitudes toward DS, including their understanding of the disease and its pharmacotherapy, as well as the provision of patient education and counseling related to this disorder in Türkiye. However, this study has certain limitations. First, only a relatively small sample of community pharmacists in Türkiye was included. The use of convenience sampling and online voluntary participation may have introduced selection bias, as pharmacists who chose to participate could differ from the broader population of community pharmacists in Türkiye in terms of interest, knowledge, or professional experience related to DS. In addition, certain geographic regions or practice settings may have been underrepresented. Second, because the survey was self-reported, there may have been a recollection bias and participants’ varying understanding of the questions. Therefore, given these limitations, the findings should be interpreted with caution and may not be fully generalizable to all community pharmacists across Türkiye. We have also emphasized that future studies using larger, randomized, and nationally representative samples are warranted to improve external validity and strengthen the generalizability of the results to a wider pharmacist population in Türkiye.

## Conclusion

This study found that Turkish community pharmacists demonstrated a good level of knowledge and positive attitudes toward DS. However, participants reported insufficient knowledge regarding pharmacotherapy and patient education and counseling related to this disorder. In this context, to enable community pharmacists to be more effectively involved in the management of this genetic condition and to enhance patient care, greater efforts are needed to improve their knowledge and awareness of DS.

## Data Availability

The original contributions presented in the study are included in the article/supplementary material, further inquiries can be directed to the corresponding author.
